# Editorial: Hippo Signaling in the Immune System

**DOI:** 10.3389/fimmu.2020.587514

**Published:** 2020-10-14

**Authors:** Lanfen Chen, Hongbo Chi, Tatsuo Kinashi

**Affiliations:** ^1^State Key Laboratory of Cellular Stress Biology, School of Life Sciences, Xiamen University, Xiamen, China; ^2^Department of Immunology, St. Jude Children's Research Hospital, Memphis, TN, United States; ^3^Department of Molecular Genetics, Institute of Biomedical Science, Kansai Medical University, Hirakata, Japan

**Keywords:** Hippo signaling, immune response, MST1/2 kinases, YAP/TAZ, NDR1/2

The Hippo signaling pathway, originally identified as a central developmental regulator of organ size, has been recently found to play indispensable roles in the immune system. In general, the canonical Hippo signaling acts through the Mst1/2-Lats1/2 kinase cassette to inhibit the activation of the transcriptional regulator YAP, and eventually inhibit cell proliferation and promote cell death during tissue development and regeneration. However, in the immune system, recent studies have found that Mst1/2 phosphorylate other substrates to activate the non-canonical Hippo signaling to regulate immune responses. In particular, patients bearing loss-of-function mutations of *MST1* (*STK4*) develop complex immunodeficiency symptoms characterized by recurrent infections, as well as autoimmune diseases. The combined phenotypes of immunodeficiency and/or autoimmunity are also observed in *Mst1* (*Stk4*)-null mice, as well as the conditional hematopoietic lineage-specific *Mst1/2* knockout mice. Thus, accumulating evidence emphasizes distinct roles of Hippo signaling in immune cells to maintain the immune homeostasis. This Research Topic aims to provide our current understanding on the roles of Hippo signaling in immunity by focusing on the cross-talk of non-canonical Hippo signaling with other classical immune signaling pathways in various immune cells under normal physiological conditions or upon the immunological insults.

In a review by Ueda et al., the authors comprehensively discuss the connection between Hippo signaling and immune responses, especially the non-canonical/alternative Hippo signaling pathway, centered with Mst1/2 kinases, in lymphocytes. Mst1/2 maintain T cell homeostasis by regulating lymphocyte development, trafficking, survival, and antigen recognition by naïve T cells. In addition, Mst1/2 orchestrate the function of regulatory T cells and effector T cells, thus acting to balance immune activation and tolerance. At the molecular levels, Mst1/2 mediate these immune functions through regulation of integrin, cytoskeleton dynamics, vesicular transports, and transcription factors. The downstream effectors of the canonical Hippo signaling, YAP and TAZ, which have been largely studied in regulating tissue development and tumor formation, are also found to be important for the function of regulatory or effector T cells in some circumstances. In an original research article, Lebid et al. report that, in addition to its pro-oncogenic role in tumor cells, YAP plays an immunoinhibitory role in CD8 T cells, especially in activated cytotoxic cells in the tumor microenvironment.

Two of the review articles in the topic are specifically dedicated to the roles of Hippo kinases and signaling in innate immunity. Zhang et al. summarize the function of the Hippo pathway in innate immune cells and how it regulates the innate anti-bacterial immunity, anti-viral immunity, as well as innate anti-tumor immunity, while Wang et al. address the crosstalk between Hippo-YAP pathway and innate immunity pathways, focusing on the IFN-I signaling, NF-κB signaling, reactive oxygen species, and the perspectives of Hippo-YAP-innate immunity crosstalk in tumor development. In non-immune cells, Lats1/2, the major downstream effectors of the canonical Hippo signaling pathway, play pivotal roles in the control of cell fate through phosphorylating and inactivating YAP. However, elimination of Mst1 in T cells has little effect on the phosphorylation of Lats1/2, while the kinases NDR1/2, members of the same family of kinases as Lats, are reported to play an important role in regulating T cell function, suggesting that Mst1/2 may activate the non-canonical Hippo signaling pathway via NDR1/2 in immune cells. In a review, Ye et al. discuss the roles of NDR1/2 in the modulation of cytokine-induced inflammation and innate immune response against the infection.

The functions of Hippo signaling in the nervous system have recently been elucidated. Microglia in the brain are the primary innate immune cells, which are essential for maintaining a local microenvironment for neuronal survival and functioning. In a review by Cheng et al., the authors summarize the recent findings pertaining to the roles and mechanisms of Hippo signaling in regulating the neuronal stem cell physiology, neuronal cell death, microglial function, neuroinflammation, and their implications in neuronal system diseases.

The manuscripts included in this Special Issue highlight and expand our understanding of the non-canonical activation of the Hippo pathway in innate and adaptive immune systems. The Hippo kinases, Mst1/2, play a central role in regulating lymphocyte development, trafficking, survival, and antigen recognition. The alternate NDR1/2 kinases modulate the inflammation response induced by cytokines and during the infection. YAP regulates the anti-viral responses and TAZ plays important roles in T lymphocyte differentiation, suggesting that YAP/TAZ, previously considered as oncogenes, are critical factors in the immune system function. These findings also highlight the Hippo signaling pathway as a potential target in infectious and autoimmune diseases, as well as raise new considerations for the current thinking on Hippo signaling as a cancer target. Thus, in order to develop the potential therapeutic strategies to fine-tune the Hippo signaling for specific disease treatment, it is important to explore deeper mechanisms of how Hippo signaling pathway regulates immune responses and tissue development.

## Author Contributions

LC, HC, and TK edited the topic. LC wrote the manuscript. HC and TK reviewed and corrected the manuscript. All authors contributed to the article and approved the submitted version.

## Conflict of Interest

The authors declare that the research was conducted in the absence of any commercial or financial relationships that could be construed as a potential conflict of interest.

